# Comparison of the pregnancy outcomes between the medical and surgical treatments in tubal ectopi pregnancy

**Published:** 2018-01

**Authors:** Azadeh Yousefnezhad, Azar Pirdehghan, Mahboubeh Roshandel Rad, Aemeh Eskandari, Shahnaz Ahmadi

**Affiliations:** 1 *Emam Khomaini Hospital, Tehran University of Medical Science, Tehran, Iran.*; 2 *Department of Community Medicine, Hamadan University of Medical Science, Hamadan, Iran.*; 3 *,Shafa Hospital, Gilan University of Medical Science, Rasht, Iran.*; 4 *,Firouzabadi Hospital, Iran University of Medical Science, Tehran, Iran.*

**Keywords:** Ectopic pregnancy, Medical treatment, Surgical treatment, Fertility, Infertility

## Abstract

**Background:**

Various treatments have been proposed to treat ectopic pregnancy, but their impact on future pregnancies is still the subject of controversy.

**Objective:**

The aim of this study is to compare the medical and surgical treatment methods and their impact on the subsequent fertility results and complications in women with a history of ectopic pregnancy.

**Materials and Methods:**

In this analytical, cross-sectional study, 370 women with the history of ectopic pregnancy, (treared with single dose of methotrexate or salpingectomy by laparotomy), that referred to Al-Zahra Hospital, Rasht, Guilan between 2009 to 2013 were enrolled. 147 women responded to the phone call. The age, the number of women that needed to drug for pregnancy, fertility rate and the fertility outcomes were studied.

**Result:**

147 women responded to the call and between them, 114 women tried to get pregnant again after the ectopic pregnancy treatment. They were agreed to the participate in the study. The mean age of the patients was 28.56±5.63 yr. The fertility rates in the medical and the surgical groups were 56.6% and 47.61%, respectively (p=0.141). There were no significant differences in the poor consequences of pregnancy among the two groups; ectopic pregnancy (p=0.605), miscarriage (p=0.605), and prematuredelivery (p=0.648). 15.1% in the medicinal group and two patients 12.5% in the surgical group had received fertility treatment in order to get pregnant (p=0.135). There was no significant difference in two groups.

**Conclusion:**

It seems that surgical treatment depending on the underlying variables of each patient, can be used such as medical treatment, without worrying about its effect on fertility.

## Introduction

A pregnancy that occurs in any place other than the endometrial cavity is called ectopic pregnancy. The incidence of ectopic pregnancy in developed countries has increased by 2% recently. Ectopic pregnancy is the most prevalent cause of mortality in pregnant women during their first trimester. It is responsible for 10% of all mortality during pregnancy. The treatment for ectopic pregnancy can have a significant effect on the health and future fertility of the patients (1). For a long time, surgical treatment had been the standard treatment for ectopic pregnancy (1). But today, medical treatment with methotrexate is the preferred treatment for patients with hemodynamically stable conditions (2). This method involves less damage to the tube, as well as lower costs, and it eliminates the risky effects of anesthesia and surgery. Various studies have reported different results in relation to the fertility status of medical and surgical treatment methods (3). There is a lack of studies in Iran that compare pregnancy outcomes in patients who were treated for ectopic pregnancy with a single dose of methotrexate and patients who were treated by the surgical procedure. This study is designed to address that gap.

## Materials and methods

This cross-sectional study analyzed the records of the patients who were admitted to the Al-Zahra Hospital with a diagnosis of ectopic pregnancy between 2009 and 2013. They were treated with either a single dose of methotrexate (50 mg/m^2^) or with the surgical (salpingectomy by laparotomy) method (4). 

The women who received surgical treatment in addition to the medicinal treatment, the women who have bilateral tubectomy during surgical treatment for ectopic pregnancy, the women who received multiple dose of methotrexate and who were cndidated for IVF or IUI in previous pregnancy, excluded from the study, other patient enrolled and were contacted by phone with them.

**Ethical consideration**

This study was approved by the Ethics Committee of the Guilan University of Medical Sciences (Code: 1910396401). Oral consent was obtained from all participants.

**Statistical analysis**

The data were analyzed with SPSS, software (Statistical Package for the Social Sciences, version 16.0 SPSS Inc., Chicago, USA). A Chi-square test was used to compare fertility and pregnancy outcomes in the two groups: those treated by medical treatment and those treated with the surgical procedure.

## Results

In our study period, 370 women were treated for tubal pregnancy in the Al-Zahra Hospital (285 with a single dose of methotrexate and 85 with the tubal surgery). Of them 147 women responded to the call and 114 women that tried to get pregnant again after the ectopic pregnancy treatment were agreed to the participate in the study. The mean age of the participitants was 28.56(±5.63) yr with a minimum age 17 and a maximum 44 years. 

Of the 114 women, 21 patients (18.4%) had been treated with surgery, while 93 patients (81.6%) had received the medicinal treatment. Out of the 114 patients, 71 (62.28%) were pregnant. Within the medicinal treatment group, 61 patients (56.6%) were pregnant, while 10 patients (47.61%) were pregnant from the surgical treatment group. The pregnancy rate in the two groups was similar and had no significant difference (p=0.141), (table I). Also, there were no significant differences in the poor consequences of pregnancy; ectopic pregnancy (p=0.605), miscarriage (p=0.605), and prematuredelivery (p=0.648) among the two groups (Table II).

A total of 14 patients (15.1%) in the medicinal group and two patients (12.5%) in the surgical group had received fertility treatment in order to get pregnant (p=0.135).

**Table I:** Fertility rate after treatment of ectopic pregnancy in two study groups

	**Medical treatment group**	**Surgical treatment group**	**p** **-value**
Pregnancy			
Yes	61 (56.6)	10 (47.6)	0.141
No	32 (34.4)	11 (52.4)	
Total	93 (100)	21 (100)	

Data presented as n (%). Fisher’s exact test.

**Table II:** Outcome of pregnancy after ectopic pregnancy treatment in two study groups

	**Medical treatment group**	**Surgical treatment group**	**p-value**
Term pregnancy	38 (62.2)	4 (40)	0.298
Preterm pregnancy	9 (14.8)	2 (20)	0.648
Abortion	7 (11.5)	2 (20)	0.605
Ectopic pregnancy	7 (11.5)	2 (20)	0.605
Total	61 (100)	10 (100)	

Data presented as n (%). Fisher’s exact test.

## Discussion

The results of this study showed that the fertility rates are the same for the two groups. This finding is similar to the results of Ergo colleagues (5), but it contradicts the findings of De Bennetot (6) and Bouyer co-workers (7). Ergo and co-workers found that the fertility rate after one year depended on factors like age, a history of infertility, and damage to the anterior wall of the tube. The fertility rate, according to them, is not related to the type of treatment administered for ectopic pregnancy (5). 

De Bennetot *et al* studied the fertility rates in 164 women who were treated for ectopic pregnancy between 1992 and 2008 with surgical and medicinal procedures (6). The study found that the percentages of intrauterine pregnancy after two years of medicinal treatment (methotrexate) and surgery (salpingectomy), respectively, were 76% and 67%. The rate of intrauterine pregnancy was higher in younger women who had been treated with the medicinal procedure. But it was found to be less in women older than 35 yr.

In our study, the pregnancy rates after one year of treatment for ectopic pregnancy in medicinal and surgical patients were 56.6% and 47.6%, respectively. Bouyer *et al.* investigated the intrauterine pregnancy rate at 18 months after treatment with methotrexate and surgical procedure and it was reported to be 80% and 57%, respectively (7).

In a study by Khazardoost and co-workers, out of the 100 patients who were treated surgically, 74% experienced intrauterine pregnancy at least three years after surgery. This can probably be explained by the long-term follow-up of the patients (8).

In our study, the incidence of ectopic pregnancy was not statistically different between the two groups. This finding was similar to the studies of Bouyer and De Bennetot colleagues (6, 7).

## Conclusion

In our study, the intrauterine pregnancy rate in patients treated with the medicinal and the surgical procedures was similar. However, the shortcomings of this study pertain to the low sample size and a short follow-up period of patients. Further study is needed to confirm or refute the findings of our study. Another limitation of our study was that laparoscopy and minimally-invasive procedures to maintain the tube were not used on any of the patients. Instead, salpingectomy was used. It is better to compare the fertility rate after medicinal therapy and non-invasive surgery by laparoscopy after an ectopic pregnancy.
